# Non-surgical endodontic retreatments in Brazil’s public dental services: a 15-year nationwide register-based study

**DOI:** 10.1590/0103-644020246004

**Published:** 2024-12-16

**Authors:** Guibson da Silva Litaiff, Ricardo Barbosa Lima, Aluísio Eustáquio de Freitas, Paulo Nelson-Filho, Raquel Assed Bezerra da Silva, Léa Assed Bezerra da Silva

**Affiliations:** 1 Graduate Program in Pediatric Dentistry, School of Dentistry of Ribeirão Preto - University of São Paulo (FORP/USP), Ribeirão Preto, São Paulo, Brazil.; 2 Department of Pediatric Dentistry, School of Dentistry of Ribeirão Preto - University of São Paulo (FORP/USP), Ribeirão Preto, São Paulo, Brazil.

**Keywords:** endodontics, retreatment, ambulatory care, COVID-19, unified health system

## Abstract

The objective of this study was to analyze the provision of non-surgical endodontic retreatments in Brazil's public dental services from 2008 to 2022. A time series was outlined for this purpose. The annual numbers of non-surgical endodontic retreatments were retrieved from the Brazilian National Outpatient Information System and adjusted per 100,000 inhabitants to obtain the incidence between 2008 and 2022. Comparison of the incidence between types of teeth, temporal trend estimation, and evaluation of the COVID-19 pandemic-related impacts were conducted with a significance level of 5%. In this timeframe, 1,270,182 non-surgical endodontic retreatments were carried out. A higher incidence was observed among single-rooted teeth (328/100,000) when compared to double-rooted (183/100,000) and multi-rooted teeth (112/100,000) (*p* <0.05). The annual incidences showed a significantly decreasing trend over the last 15 years for all types of teeth (*p* <0.05), as well as demonstrated a highly correlated pattern of temporal variation (*p* <0.05). In addition, there was no influence on this outcome when removing the period between 2020 and 2022 (all temporal trends remained decreasing). However, the monthly incidence was significantly lower in the first, second, and third years after the COVID-19 pandemic onset (*p* <0.05). The provision of non-surgical endodontic retreatments in Brazil's public dental services has experienced a dramatic decline over the past 15 years, including after the COVID-19 pandemic.

## Resumo

O objetivo deste estudo foi analisar a provisão de retratamentos endodônticos não-cirúrgicos nos serviços odontológicos públicos do Brasil entre 2008 e 2022. Foi construída uma série temporal com esse propósito. Os números anuais de retratamentos endodônticos não-cirúrgicos foram obtidos do Sistema de Informações Ambulatoriais do Brasil e ajustados a cada 100.000 habitantes para obter a incidência entre 2008 e 2022. A comparação da incidência entre tipos de dentes, a estimativa da tendência temporal e a avaliação dos impactos relacionados à pandemia da COVID-19 foram realizadas com um nível de significância de 5%. Neste período, foram realizados 1.270.182 retratamentos endodônticos não-cirúrgicos. Foi observada uma incidência maior entre os dentes unirradiculares (328/100.000) quando comparados aos dentes birradiculares (183/100.000) e com três ou mais raízes (112/100.000) (*p* <0,05). As incidências anuais apresentaram uma tendência significativamente decrescente nos últimos 15 anos para todos os tipos de dentes (*p* <0,05), além de exibirem um padrão altamente correlacionado de variação temporal (*p* <0,05). Ademais, não houve influência nesse resultado ao remover o período entre 2020 e 2022 (todas as tendências temporais permaneceram decrescentes). No entanto, a incidência mensal foi significativamente menor no primeiro, segundo e terceiro ano após o início da pandemia de COVID-19 (*p* <0,05). A provisão de retratamentos endodônticos não-cirúrgicos nos serviços odontológicos públicos do Brasil experimentou uma queda dramática nos últimos 15 anos, incluindo após a pandemia da COVID-19.

## Introduction

Brazil's public healthcare system, also known as the Unified Health System (*Sistema Único de Saúde* or SUS), is one of the largest providers of healthcare worldwide, including public dental services. Indeed, the majority of Brazilians rely exclusively on public initiative to address their healthcare needs, highlighting the significance of its productivity in this country ^1^ ^,^ ^2^. For oral health, public dental services are distributed throughout the national territory, mainly in Primary Health Care (PHC; such as oral health teams acting in the Family Health Strategy) and Secondary Health Care (SHC; such as Dental Specialty Centers - DSCs). Oral health care in this system is based on PHC for primary demands, with low technological density, as well as referrals to SHC for more complex cases (specialized dental care), both providing outpatient dental care for Brazilians ^3^ ^,^ ^4^.

Considering the oral public health care in Brazil, it is also important to highlight the significance of Endodontics as a dental specialty available within this system, emphasizing the contribution of all endodontic treatments in facing unnecessary tooth extractions and investing in restorative and rehabilitative strategies that prevent tooth loss. This perspective is crucial for Collective Oral Health, as well as for outpatient productivity analysis within a public healthcare system that aims to improve oral health indicators among its population ^4^ ^,^ ^5^. Addressing this topic, two recent studies aimed to investigate outpatient productivity in Brazil's public healthcare system, including endodontic-related outcomes. The first study covered the period from 1999 to 2017 ^6^ and the second from 2008 to 2018 ^7^.

In the first study, endodontic treatments represented 0.5% of approximately 3.5 billion outpatient dental procedures carried out during 19 years, and the temporal trend was defined as stationary for the national estimate ^6^. Stationarity in the provision of endodontic treatments was also observed in the second study, considering approximately 2.6 billion dental procedures over 11 years ^7^. However, there was no differentiation between endodontic treatments (first root canal obturation - pulpectomy) and non-surgical retreatments in their approaches. The productivity of Endodontics as a specialty was estimated for both modalities.

From this analysis, a gap in the state of the art is considered, as properly evaluating non-surgical endodontic retreatments would be valuable for a deep understanding of the dynamics surrounding the provision of all endodontic treatments within Brazil's public healthcare system. This statement is based on the possibility of failures in endodontic treatments (despite significant advances in clinical success rates in recent years), which does not immediately imply the need for tooth extraction, as long as retreatment is indicated and feasible (thus avoiding tooth loss and the need for rehabilitation through more invasive and costly strategies, such as dental implants) ^8^ ^,^ ^9^.

The context of Endodontics and endodontic retreatments should also be viewed from the perspective of the COVID-19 pandemic, as the outbreak of SARS-CoV-2 in 2020 and the sanitary measures to contain its transmission have negatively impacted the provision of outpatient dental procedures within Brazil's public healthcare system. Two previous studies, comparing 2019 (control interval) and 2020, demonstrated the impact of the COVID-19 pandemic on the provision of outpatient dental procedures, both elective (non-urgent) and urgent ^10^, as well as endodontic-related ones ^11^. However, similar to the context discussed earlier, endodontic treatments and non-surgical retreatments were not differentiated, in addition to occurring within a limited period at the onset of the COVID-19 pandemic (only the first year).

Taking all the presented evidence into account, it is reasonable to consider that conducting an analysis of outpatient productivity focused on non-surgical endodontic retreatments may be an important step in understanding the national context, including a before-and-after perspective of the COVID-19 pandemic. Therefore, the aim of this study was to analyze the provision of non-surgical endodontic retreatments in Brazil's public dental services from 2008 to 2022, exploring the person-year incidence, temporal trend, and COVID-19 pandemic impacts. Hence, three null hypotheses were examined: H_0_1 - The incidence of non-surgical endodontic retreatments was similar among single-rooted, double-rooted, and multi-rooted teeth; H_0_2 - There was a stationary temporal trend in the annual incidence of non-surgical endodontic retreatments, and H_0_3 - There was no significant impact of the COVID-19 pandemic on the provision of non-surgical endodontic retreatments.

## Materials and Methods

### Study Design

For this purpose, a nationwide register-based time series was delineated, as an epidemiological, longitudinal, retrospective, and quantitative study, similar to previous studies ^6^ ^,^ ^7^. The spatial component was delimited to Brazil, considering the nationwide outpatient productivity within Brazil's public healthcare system. The temporal component was delimited from 2008 to 2022 (annual data), considering a 15-year analysis. Regarding the types of dental public health services, no restriction was applied, considering all non-surgical endodontic retreatments financed by this system. In addition, a specific design was conducted to assess the impact of the COVID-19 pandemic (monthly data), considering a preceding interval as control (from April 2019 to March 2020) and the three subsequent years after its onset (from April 2020 to March 2023, respectively), also similar to previous studies ^2^ ^,^ ^5^.

### Ethics

All data were obtained from sources accessible in the public domain (open access), ensuring adherence to ethical standards in research with a register-based approach that involves no direct interaction with human subjects. Therefore, in accordance with Resolution 510, issued on April 7, 2016, by the National Health Council - Ministry of Health, ethical approval by a Research Ethics Committee is not required (Article 1, sole paragraph, items II, III, and V) ^12^.

### Data Source

The primary data source was the National Outpatient Information System (SIA/SUS). This data source is managed by the Department of Informatics of Brazil's public healthcare system (DATASUS) and provides the monthly/annual numbers of all outpatient dental procedures carried out across the entire Brazilian territory (notified through Outpatient Productivity Bulletins - OPBs) ^13^. In addition, Brazilian intercensal population projections were utilized, considering the annual estimates from the Brazilian Institute of Geography and Statistics (IBGE).

### Variables

The primary variable of the study was the incidence of endodontic retreatments (non-surgical approach). Monthly/annual quantities were normalized per 100,000 inhabitants to correct for the effect of demographic changes over time. In the SIA/SUS, these dental procedures are reported after the completion of root canal obturation using the codes #03.07.02.010-0 (for single-rooted teeth), #03.07.02.008-8 (for double-rooted teeth), and #03.07.02.009-6 (for multi-rooted teeth). As secondary variables, the ratio between endodontic treatments (first root canal obturation) and non-surgical retreatments (T/R ratio) was examined for each type of tooth (single-, double-, and multi-rooted), as well as the ratio between tooth extractions and non-surgical endodontic retreatments (E/R ratio) for all types of teeth. The treatments were retrieved similarly to the retreatments, according to the types of teeth, using the codes #03.07.02.006-1, #03.07.02.004-5, and #03.07.02.005-3, respectively. Tooth extractions, on the other hand, were retrieved using a sole code, #04.14.02.013-8.

### Data Collection

The data collection was conducted between November and December 2023 by a sole researcher with experience in utilizing the mentioned data sources. On the DATASUS website, the TabNet tool was used to access data originating from the SIA/SUS. The available filters were used to adjust the spatial (Brazil) and temporal (from 2008 to 2022) components, as well as to specify the outpatient dental procedures of interest to the study. For productivity purposes, solely non-surgical endodontic retreatments approved by Brazil's public healthcare system were considered. After the collection, the data were exported and stored in spreadsheets for subsequent analysis. The data collection procedures described are similar to those adopted in previous studies ^5^ ^,^ ^6^ ^,^ ^7^.

### Data Analysis

The data were analyzed at a significance level of 5% (α = 0.05), considering *p*-values <0.05 as statistically significant. PAST (version 4.3, Oslo, Norway) statistical package was employed for temporal trend analyses, and JAMOVI (version 2.3.15, Sydney, Australia) was utilized for data description, correlations, and comparison analyses. Descriptively, in addition to the incidence for the period (number of dental procedures/number of inhabitants × 100,000), the median of annual incidences between 2008 and 2022, including the interquartile range (IQR) and the minimum and maximum values, was obtained. In addition, the T/R and E/R ratios were obtained by simple division between the absolute frequencies in each year.

The correlations were examined using the Spearman correlation matrix, in which the *rho* coefficient (ρ) indicated the direction and strength. Since the primary/dependent variable was based on count data (integers), generalized linear models were used to compare the incidences. Considering a quasi-Poisson distribution with overdispersion (robust variance), Negative Binomial regression analysis was employed. Incidence ratios (IR) were estimated using maximum likelihood in the logarithmic link function. The trend over time was expressed by the Annual Percent Change (APC), considering the estimation of angular coefficients (β_1_; slopes) and coefficients of determination (R^2^) using Prais-Winsten regression analysis after a logarithmic transformation (*log*10) of the dependent variables, as previously described. All trends were established as stationary (*p* ≥0.05), decreasing (*p* <0.05 and negative β_1_), or increasing (*p* <0.05 and positive β_1_) ^14^.

## Results

Over the past 15 years, 1,270,182 non-surgical endodontic retreatments were carried out in Brazil's public dental services across the entire national territory. Single-rooted teeth were the most frequent, comprising approximately 52.6% (668,266), accompanied by double-rooted teeth at 29.4% (373,299), and multi-rooted teeth at 18% (228,617). Table 1 describes the incidence of endodontic retreatments for all types of teeth. It was noted that the lowest incidence occurred in 2020 for all types of teeth, while the highest incidence was recorded in the first year of the time series (2008). In addition, the highest incidence was observed among single-rooted teeth from 2008 to 2022.

**Table 1:** Incidence of non-surgical endodontic retreatments for all types of teeth per 100,000 inhabitants from 2008 to 2022 (2024).

Types of teeth	Incidence	Annual incidence (median)	IQR	Minimum (year)	Maximum (year)
Single-rooted	328/100,000	17/100,000	26	4 (2020)	70 (2008)
Double-rooted	183/100,000	15/100,000	10	4 (2020)	24 (2008)
Multi-rooted	112/100,000	6/100,000	5	4 (2022)	16 (2008)

IQR: interquartile range (Q3 - Q1).

Moreover, Table 2 presents the appropriate comparison of the incidence of non-surgical endodontic retreatments among all types of teeth. The single-rooted teeth were used as the reference level due to their highest incidence. In relation to them, the incidence of non-surgical endodontic retreatments in double-rooted ones was 44.5% lower (95% CI = 12.4, 64.9), while in multi-rooted teeth it was 66.2% lower (95% CI = 45.8, 78.9) from 2008 to 2022 within Brazil’s public healthcare system.

**Table 2:** Incidence of non-surgical endodontic retreatments among all types of teeth (2024).

Comparison	IR	Limits		*p*
		Lower	Upper	
Intercept	12.8	10.6	15.6	<0.001*
Single-rooted	*ref*			
Double-rooted	0.555	0.351	0.876	0.012*
Multi-rooted	0.338	0.211	0.542	<0.001*

IR: incidence ratio. *ref*: reference level (IR = 1). *: *p* <0.05 (statistically significant).

**Figure 1:**
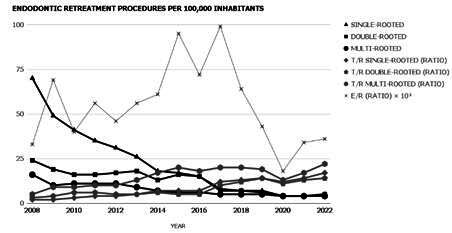
Temporal variation of the incidence of non-surgical endodontic retreatments from 2008 to 2022 within Brazil’s public healthcare system.

Considering the secondary variables, the T/R ratio was estimated at 4.6 for single-rooted teeth, 6.2 for double-rooted teeth, and 13 for multi-rooted teeth, while the E/R ratio was estimated at 535 over the last 15 years. Table 3 presents the temporal trend of the incidence of non-surgical endodontic retreatments for all types of teeth, including a secondary analysis predating the COVID-19 pandemic (from 2008 to 2019), as well as T/R and E/R ratios. Figure 1 demonstrates the temporal variation of the incidence of non-surgical endodontic retreatments from 2008 to 2022 within Brazil’s public healthcare system, also including T/R and E/R ratios. It is noteworthy that there was a significant, positive, and very strong correlation in the annual incidence of non-surgical endodontic retreatments between single-rooted and double-rooted teeth (*p* <0.001, ρ = 0.940), between single-rooted and multi-rooted teeth (*p* <0.001, ρ = 0.968), and between double-rooted and multi-rooted teeth (*p* <0.001, ρ = 0.908).

**Table 3:** Temporal trend of the incidence of endodontic retreatments for all types of teeth per 100,000 inhabitants from 2008 to 2022 (2024).

Timeframe /Types of teeth	β_1_	R^2^	*p*	Trend	APC (%)
2008 - 2022					
Single-rooted	-0.090	0.975	<0.001*	Decreasing	-18.7
	[-0.098, -0.081]				[-20.2, -17.0]
Double-rooted	-0.054	0.839	<0.001*	Decreasing	-11.7
	[-0.067, -0.041]				[-14.3, -9.0]
Multi-rooted	-0.042	0.912	<0.001*	Decreasing	-9.2
	[-0.050, -0.034]				[-10.9, -7.5]
T/R ratio	0.071	0.949	<0.001*	Increasing	17.8
(single-rooted)	[0.058, 0.079]				[14.3, 19.9]
T/R ratio	0.046	0.797	<0.001*	Increasing	11.2
(double-rooted)	[0.033, 0.055]				[7.9, 13.5]
T/R ratio	0.037	0.690	<0.001*	Increasing	8.9
(multi-rooted)	[0.016, 0.046]				[3.8, 11.2]
E/R ratio	-0.007	0.052	0.411	Stationary	-1.6
	[-0.040, 0.015]				[-8.8, 3.5]
2008 - 2019					
Single-rooted	-0.091	0.973	<0.001*	Decreasing	-18.9
	[-0.100, -0.081]				[-20.6, -17.0]
Double-rooted	-0.049	0.748	<0.001*	Decreasing	-10.7
	[-0.070, -0.031]				[-14.9, -6.9]
Multi-rooted	-0.045	0.888	<0.001*	Decreasing	-9.8
	[-0.055, -0.033]				[-11.9, -7.3]
T/R ratio	0.082	0.971	<0.001*	Increasing	20.8
(single-rooted)	[0.074, 0.093]				[18.6, 23.9]
T/R ratio	0.053	0.690	<0.001*	Increasing	13
(double-rooted)	[0.032, 0.073]				[7.6, 18.3]
T/R ratio	0.048	0.861	<0.001*	Increasing	11.7
(multi-rooted)	[0.030, 0.061]				[7.2, 15.1]
E/R ratio	0.019	0.208	0.137	Stationary	4.5
	[-0.012, 0.042]				[-2.7, 10.2]

β_1_: angular coefficient (slope). R^2^: coefficient of determination. APC: Annual Percent Change. *: *p* <0.05 (statistically significant). [ ]: 95% confidence interval. T/R: treatment/retreatment. E/R: extraction/retreatment.

It was observed that there was a significant decreasing trend in the incidence of non-surgical endodontic retreatments in all types of teeth, with high R^2^ values. The COVID-19 pandemic did not affect this outcome after removing the interval between 2020 and 2022 (with the exception of slight changes in the R^2^ values). Considering the T/R ratio, there was a significant increasing trend and moderate-to-high R^2^ values. Similarly, the COVID-19 pandemic did not affect this outcome. Furthermore, the E/R ratio remained stationary in both timeframes (from 2008 to 2022 and from 2008 to 2019). However, there was a change in the sign of the angular coefficient (from negative to positive) and an increase in the R^2^ value after removing the interval between 2020 and 2022. In addition, it is important to note that 134,337 non-surgical endodontic retreatments were carried out in 2008, contrasting with 15,463 in 2019 (prior to the COVID-19 pandemic) and 9,841 in 2022 (after the COVID-19 pandemic onset), considering all types of teeth.

Table 4 presents the appropriate comparison of the incidence of non-surgical endodontic retreatments before and after the COVID-19 pandemic onset. It was observed that there was a significant decrease in monthly incidences compared to the control period, estimated at 48.4% lower (95% CI = 33.9, 59.7) in the first year, 27.7% lower (95% CI = 9.7, 42.2) in the second year, and 27.2% lower (95% CI = 9.0, 41.7) in the third year.

**Table 4:** Incidence of endodontic retreatments before and after the COVID-19 pandemic onset (2024).

Comparison	IR	Limits		*p*
		Lower	Upper	
Intercept	1.11	1.02	1.21	<0.001*
Pre-pandemic	*ref*			
First year	0.516	0.403	0.661	<0.001*
Second year	0.723	0.578	0.903	0.004*
Third year	0.728	0.583	0.910	0.005*

IR: incidence ratio. *ref*: reference level (IR = 1). *: *p* <0.05 (statistically significant).

## Discussion

Considering the three null hypotheses examined, H_0_1 was rejected, as the incidence of non-surgical endodontic retreatments in single-rooted teeth was significantly higher compared to double-rooted and multi-rooted teeth. H_0_2 was also rejected, as the annual incidence of non-surgical endodontic retreatments showed a significantly decreasing temporal trend over the last 15 years for all types of teeth (even after removing the interval between 2020 and 2022 due to the COVID-19 pandemic onset). Likewise, H_0_3 was rejected, as the monthly incidence of non-surgical endodontic retreatments after the COVID-19 pandemic onset was significantly lower during the first, second, and third subsequent years.

The significant decrease in the annual incidence of non-surgical endodontic retreatments in recent years is a complex outcome. Firstly, it is important to consider the advancements in radical endodontic therapies in recent years, achieving high rates of clinical success over time, which may have impacted the necessity for retreatments to some extent. Enhancements in operative techniques and microbial control strategies, for instance, can be cited as significant factors contributing to the high resolution of contemporary Endodontics ^15^ ^,^ ^16^. However, for such a dramatic decline, it was necessary to explore other important pillars in the provision of outpatient dental procedures in Brazil's public dental services.

In a historical context, it was not until 2004 that endodontic treatment (including retreatments) obtained regulation and funding from the public sector in Brazil. As relatively expensive therapeutic modalities, specialized professionals and specific dental materials are required, which results in significant costs. However, there is no doubt that endodontic treatments/retreatments should always be considered, avoiding tooth extractions ^17^. Despite the high financial impact on the healthcare system, Endodontics remains one of the most common dental specialties in the SHC within Brazil’s public dental services. Nevertheless, the waiting time to access it can be relatively long (more than 20 days after referral from the PHC), especially for patients experiencing clinical discomfort or pain ^17^ ^,^ ^18^. Thus, it is necessary to address the decrease in non-surgical endodontic retreatments from these perspectives: Is public funding adequate for this therapeutic modality? Are they resorting to tooth extractions or private dental services after the failure of the first root canal treatment?

In 2007, 49.1% of the DSCs did not meet productivity targets in Endodontics in Brazil. In this study, the authors contribute to the perspective that the provision of dental specialties in these SHC-linked dental services should be demand-driven, which could favor the achievement of goals ^19^, a perspective that corroborates the previously raised concerns regarding endodontic-related dental procedures in Brazil's public dental services. In addition, considering a more recent study covering the period between 2008 and 2018, Endodontics showed a decreasing temporal trend in the proportion of DSCs that met the goals set for this specialty (APC = -1.27% (95% CI = -2.53, -0.02) in the national estimate). This analysis took into account both treatments and retreatments ^20^.

Considering the decreasing trend of non-surgical endodontic retreatments and the increasing trend of the T/R ratio, it is important to examine the impact of this decrease on productivity indicators, both in DSCs ^19^ ^,^ ^20^ and in national outpatient analyses ^6^ ^,^ ^7^. Removing non-surgical endodontic retreatments from these indicators might show better outcomes, reflecting the variations in the provision of these therapeutic modalities over time. Additionally, the outcomes related to the E/R ratio are noteworthy. The significant decrease in non-surgical endodontic retreatments led to the stationarity of the E/R ratio, influenced by the fluctuating pattern of tooth extractions in Brazil's public dental services, with sharp annual changes over the past 15 years, despite a notable decline between 2017 and 2020.

Furthermore, analyzing non-surgical endodontic retreatments provided insights into a distinct landscape compared to previous understandings, revealing specific patterns of incidence and temporal trends. It is important to note that the provision of endodontic procedures depends on more than just the availability of dental services or oral health professionals. Factors such as user demand, management of financial and human resources, PHC coverage, availability of proper technologies and dental materials, access and duration of treatment, and the frequency of use of public and private dental services all influence the provision of these procedures. Therefore, the estimated incidence and temporal trends are complex outcomes resulting from the interaction of these factors ^6^ ^,^ ^20^ ^,^ ^21^.

An additional significant finding of this study was the higher incidence of non-surgical endodontic retreatments in single-rooted teeth when compared to double- and multi-rooted ones. Moreover, this type of tooth also showed the lowest T/R ratio. This was an interesting outcome, as there is no evidence supporting factors that would suggest a higher failure rate in single-rooted teeth after endodontic treatments, such as pulpectomy, compared to double- or multi-rooted ones. Instead, there is evidence of a lower success rate among molars and premolars when compared to incisors or canines. Indeed, risk factors for the failure of root canal therapies are more closely associated with microbiological and operative aspects ^15^ ^,^ ^22^.

Furthermore, it is conceivable that dental services linked to Brazil's public healthcare system may adopt priority criteria in Endodontics (including endodontic retreatments), categorizing patients requiring intervention in anterior teeth, such as incisors and canines, as a high priority. Conversely, for multi-rooted teeth, factors such as the need for subsequent oral rehabilitation, the involvement of the dental element in dental prostheses, losses of other dental elements in the same arch, and the degree of coronal destruction are also considered, which may limit the provision of endodontic retreatments in this type of tooth ^21^.

Concerning the COVID-19 pandemic, this outcome suggests both an immediate and late impact of the pandemic-related context on the provision of non-surgical endodontic retreatments within Brazil’s public healthcare system. However, it is crucial to note that this phenomenon cannot be disassociated from the outcomes related to the entire time series (temporal trend analysis), as the decreasing tendency persisted even after excluding the interval between 2020 and 2022. Nevertheless, the SARS-CoV-2 outbreak immediately impacted the clinical routines of this dental specialty, restricting therapies and prioritizing approaches that do not generate aerosols to reduce the risk of contamination. In fact, the COVID-19 pandemic acted as yet another stressor in endodontic practice ^23^ ^,^ ^24^.

As limitations, it is important to note that researchers do not interfere at any stage of the acquisition and management of the information available in the data source. Therefore, it is possible that some degree of misreporting may have occurred. Furthermore, outpatient productivity may vary among Brazilian macro-regions or smaller health regions, and this perspective is important due to Brazil's territorial dimension and its disparities. At last, slight variations in the monthly/annual numbers may occur over time. The state of the art still lacks new studies on this topic, including a deeper investigation into local dynamics related to non-surgical endodontic retreatments.

The provision of endodontic retreatments occurred in every year of the time series. However, a dramatic decline has been observed in the provision of non-surgical endodontic retreatments in Brazil's public healthcare system over the past 15 years, particularly after the COVID-19 pandemic. This decline was observed among all types of teeth, although a homogeneous pattern of temporal variation was observed among them from 2008 to 2022.
